# Appearance of Hürthle cell carcinoma soon after surgical extirpation of Hürthle cell adenoma and follicular adenoma of the thyroid gland

**DOI:** 10.2478/raon-2014-0047

**Published:** 2015-03-03

**Authors:** Nevena Ristevska, Sinisa Stojanoski, Daniela Pop Gjorceva

**Affiliations:** Institute of Pathophysiology and Nuclear Medicine, Acad. “Isak S. Tadzer”, Skopje, Macedonia

**Keywords:** thyroid, Hürthle cell carcinoma, follicular adenoma, Hürthle cell adenoma, ^99m^Tc-MIBI scintigraphy, radioiodine therapy

## Abstract

**Background:**

Hürthle cell neoplasms could be benign (Hürthle cell adenoma) or malignant (Hürthle cell carcinoma). Hürthle cell carcinoma is a rare tumour, representing 5% of all differentiated thyroid carcinomas. The cytological evaluation of Hürthle cell neoplasms by fine needle aspiration biopsy (FNAB) is complicated because of the presence of Hürthle cells in both Hürthle cell adenoma and Hürthle cell carcinoma. Thus, the preoperative distinction between these two entities is very difficult and possible only with pathohistological findings of the removed tumour.

**Case report:**

A 57-year old female patient was admitted at our Department, for investigation of nodular thyroid gland. She was euthyroid and FNAB of the nodules in both thyroid lobes were consistent of Hürthle cell adenoma with cellular atypias. After thyroidectomy the histopathology revealed Hürthle cell adenoma with high cellular content and discrete cellular atypias in the left lobe and follicular thyroid adenoma without cellular atypias in the right lobe. One year after substitution therapy, a palpable tumour on the left side of the remnant tissue was found, significantly growing with time, presented as hot nodule on ^99m^Tc-sestamibi scan and conclusive with Hürthle cell adenoma with marked cellularity on FNAB. Tumorectomy was performed and well-differentiated Hürthle cell carcinoma detected. The patient received ablative dose of 100 mCi ^131^I. No signs of metastatic disease are present up to date.

**Conclusions:**

The differences between Hürthle cell adenomas and Hürthle cell carcinomas could be clearly made only by histopathological evaluation. Patients with cytological diagnosis of Hürthle cell neoplasms should proceed to total thyroidectomy, especially if tumour size is > 1cm, FNAB findings comprise cellular atypias and/or multiple bilateral nodules are detected in the thyroid gland.

## Introduction

Hürthle cell neoplasms (HCN) could be of benign appearance (Hürthle cell adenoma - HCA) or the cells could undergo malignant transformation (Hürthle cell carcinoma - HCC). Hürthle cell carcinomas are rare thyroid tumours, representing about 5% of all differentiated thyroid carcinomas. They originate from the follicular cells of the thyroid gland and in 75% are composed of so called oncocytes or Hürthle cells. These cells were described for the first time by Askanazy, as cells with polygonal shape, acidophilic granular cytoplasm, hyperchromatic nuclei and abundant mitochondria.^1^ Being more aggressive than the papillary and follicular variant, Hürthle cell carcinomas should take an important part in the diagnostic and therapeutic guidelines for thyroid carcinomas. The cytological evaluation of HCN by fine-needle aspiration biopsy (FNAB) is complicated because of the presence of Hürthle cells in both – HCA and HCC. Thus, the preoperative distinction between these two entities is very difficult and possible only with pathohistological findings of the surgically removed tumours, based on identification of capsular or vascular invasion, or the presence of metastatic disease.^2^

## Case report

A 57-year old female patient was admitted at our Department for investigation of enlarged and nodular thyroid gland in February 2009, noticed firstly by her general practitioner. Written informed consent of patient was obtained for the treatments and for the scientific use of the clinical data according to Declaration of Helsinki.

**FIGURE 1.**
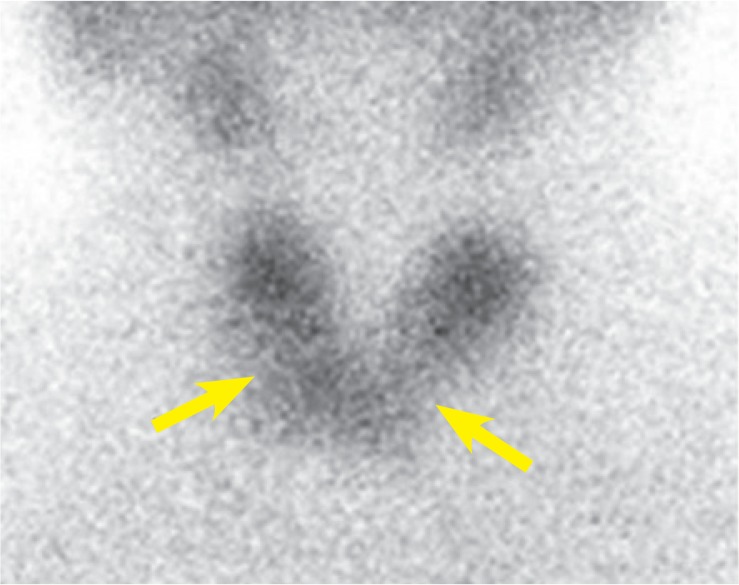
^99m^TcO4 scan showing “cold” nodules in the both thyroid lobes.

The patient was euthyroid, had no local or systemic complains, with thyroid functional tests within normal range (FT4 = 17.7 mmol/L, TSH = 1.36 IU/l). Nodules in both thyroid lobes were detected by palpation - smaller one in the right lobe and bigger one in the left lobe. Ultrasound (US) revealed isoechoic, non-homogenic nodule with hypoechoic halo (10 × 12 × 16 mm) in the middle part of the right thyroid lobe and smaller hyperechoic zone above it (7 × 4 × 8 mm). The nodule located in the lower 2/3 of the left lobe was hypoechoic, non-homogenic with cystic degeneration (20 × 27 × 39 mm). A scintiscan was performed, 20 min after intravenous application of 74 MBq of ^99m^TcO_4,_ that showed reduced uptake of ^99m^TcO_4_ in the nodule in the middle of the right lobe and a “cold” nodule in the lower 2/3 of the left lobe (Figure 1).

The FNAB of the right thyroid nodule (located in the middle, measured 16mm) detected benign Hürthle cells with abundant basophilic cytoplasm, normochromatic nucleuses and big nucleolus in central position. Some of these cells showed cytological atypias. Hürthle cells with some cytological atypias were also noted by FNAB, in the left thyroid nodule. Both FNAB findings were consistent of Hürthle cell adenomas with cellular atypias.

**FIGURE 2.**
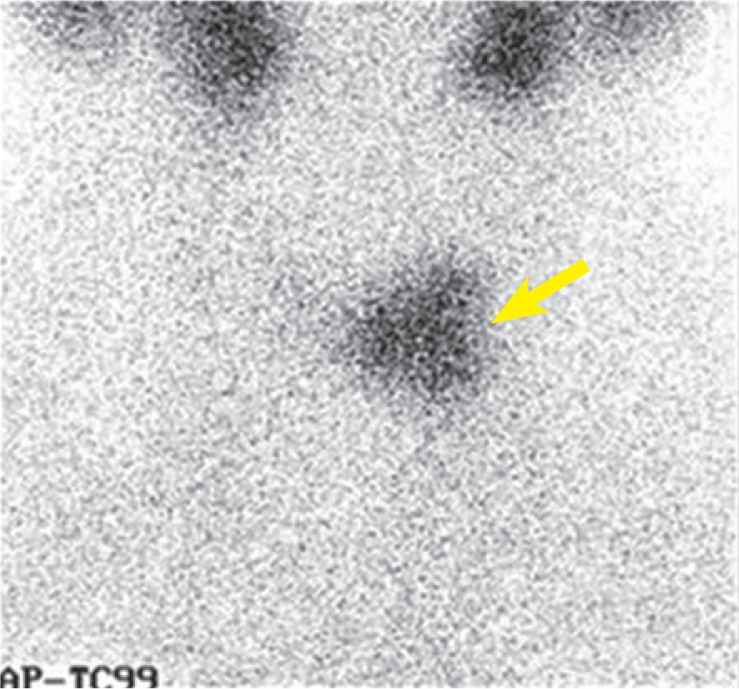
^99m^TcO4 scan showing remnant thyroid tissue on the left side after the first operation.

Because of these findings, total thyroidectomy was suggested. Near total thyroidectomy was performed (November 2009). The extent of the intervention included: *Lobectomia subtotalis l. sin*; *Lobectomia l. dext* and *Isthmectomia subtotalis*. Histopathology revealed HCA with high cellular content and discrete cellular atypias in the nodule, located in the surgically removed tissue fragment (6 × 4.5 × 3.2 cm) of the left lobe and follicular thyroid adenoma without cellular atypias in the nodule located in the surgically removed tissue fragment (4 × 3 × 2.8 cm) of the right lobe. No evidence of malignant cell transformation or capsular invasion was detected. One month after surgery (December 2009), scintiscan with ^99m^TcO_4_ showed remnant thyroid tissue (24 mm, isoechoic, non-chomogenic oval formation) on the left side, towards isthmic location and no remnant tissue on the right side of the thyroid bed (Figure 2).

Because of the hypothyroid state (FT4 = 5.11 mmol/L, TSH = 36.9 IU/l), substitution therapy with L-thyroxin was administered. Since there was no indication of malignant transformation or capsular invasion on histopathology, removal of the remnant thyroid tissue was not suggested. The size and structure of the remnant tissue on the following regular check-ups was evaluated by US (17 × 11 × 24, isoechoic non-chomogenic structure), without enlargement or nodular presentation on US on the first two regular check-ups. Thereafter, the patient was euthyroid with laboratory findings within normal range.

**FIGURE 3.**
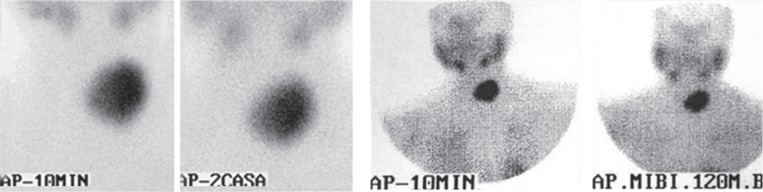
^99m^Tc-MIBI scan showing intensive accumulation in the newly appeared tumour in the left thyroid lobe.

Almost one year after surgery (patient hasn’t come for regular check-ups), at September 2010, a palpable tumour on the side of the remnant thyroid tissue (left side) was found, that showed significant progressive enlargement on US with time (from 22 mm, to 28 mm, to 39 mm). FNAB (April 2011) detected Hürthle cells with big nucleus, big acidophilic nucleolus and poor cytoplasm, cytologically conclusive of HCA with marked cellularity. To evaluate mitochondrial activity and tumour avidity, as a marker of potential malignancy, the double phase ^99m^Tc- sestamibi (MIBI) scintigraphy was performed. Intensive accumulation was noted in this nodule on the early scintigram (10 min), without washout of the radiotraser on the late (2h) phase (Figure 3).

**FIGURE 4.**
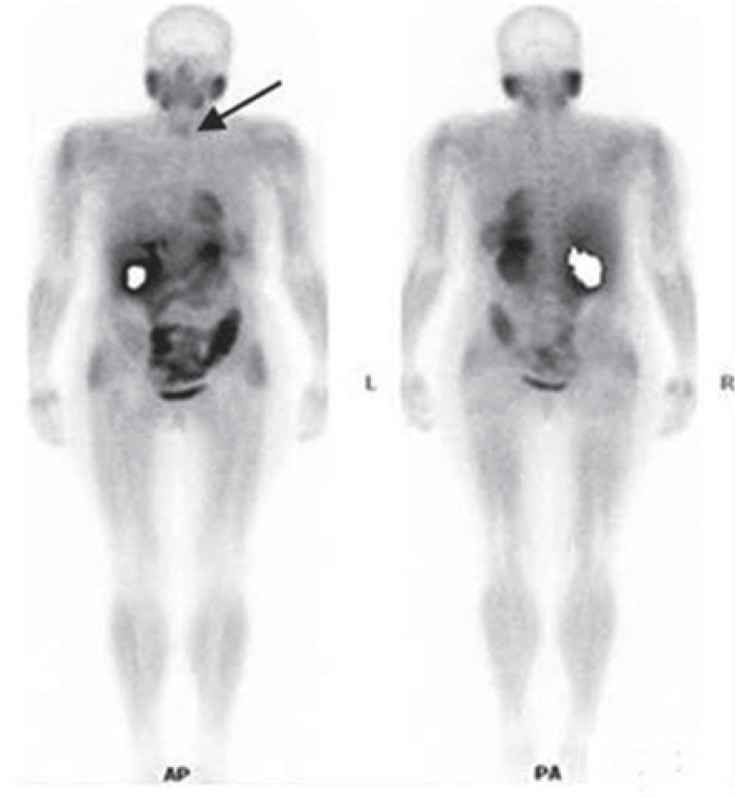
Whole-body scan (WBS) with ^99m^Tc-MIBI showing normal distribution of the tracer in the body as well as the accumulation in the tumor in the left thyroid lobe.

Considering the FNAB findings, ^99m^Tc-MIBI scan and tumour size (increasing with time) the patient was suggested and underwent a second operation (August 2012) - tumourectomy and remnant tissue extirpation. Hystopatological examination of the extirpated tissue fragment - (comprising the newly detected nodule and the remnant thyroid tissue, sized 5.5 × 4.5 × 4 cm) revealed presence of well-differentiated HCC (stage III, pTNM = pT3 pNx pMx, G1 C1) with large malignant cells rich with eosinophilic cytoplasm, with hyperchromatic nucleuses, and well-differentiated grade 1 nucleus. Necrotic regions and capsular invasion were found as well. US postoperatively revealed only small remnant thyroid tissue on the left side, confirmed on the consecutive ^99m^Tc-MIBI scintigraphy (January 2013) (Figure 4).

**FIGURE 5.**
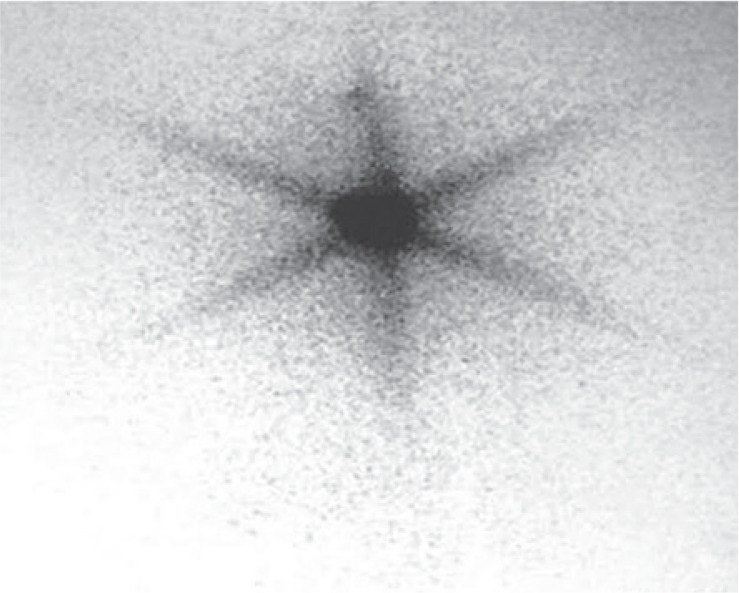
Post-therapy whole-body scan (WBS) - after ablative dose of radioiodine - showing uptake only in the thyroid bad.

One month after, an ablative dose of 100 mCi ^131^I was given and there was no pathological accumulation of the tracer on the whole-body scan (WBS), except the small accumulation in the region of the remnant thyroid tissue after the second surgical procedure (Figure 5). The patient at present is symptom free, receiving hormonal replacement therapy. No signs of metastatic disease are present up to date and the levels of thyroglobulin (Tg) are within normal range - respectively < 0.2 ng/ml.

## Discussion

Hürthle cell carcinomas are oxyphilic type of tumours, with histological features and ability of thyroglobulin production, which suggests that they arise from the follicular cells of the thyroid gland. In the beginning they were considered as a variant of the follicular thyroid carcinomas, but later the morphological findings suggested that they are separate pathological entity. Cellular features on light microscopy include large size, polygonal to square distinct cell borders, voluminous granular and eosinophilic cytoplasm, large hyperchromatic nucleus and cherry pink nucleoli.^3^ Electron microscopy reveals a granular cytoplasm due to large number of mitochondria within the Hürthle cells.^4^

HCCs comprise 2% to 10% of all differentiated thyroid carcinomas and are usually presented in the fifth to seventh decade of life.^5^–^7^ They are mainly slow-growing thyroid nodules, associated with lymphadenopathy, vocal cord paralysis and distant metastasis (usually uncommon). In 15% to 35% of cases, HCC are presented as multifocal and in up to 20% of cases lymph node metastases are presented in the beginning of the disease. About 10% of the metastases from HCCs concentrate iodine, comparing to 75% of the metastases from the follicular carcinomas.^8^

Apart from the findings of other authors, Pu *et al*. in their paper concluded that a diagnosis of HCN does not impart a higher rate of malignancy than a diagnosis of follicular neoplasm by FNAB of thyroid nodules. They also confirm that factors as male gender and older age in general are associated with worse outcome, especially with HCC.^9^

Diagnosis of HCC can be confirmed by histopathology examination of the specimen, through determination of invasiveness of the carcinoma and not only by cytological examination.^10^ The verification of invasiveness is based on demonstration of capsular invasion or angio-invasion that can be assessed only in the resected specimen.^11^ Furthermore, findings of local invasion, lymph node spread and distant metastases classify HCN as a malignant one. But aside from capsular and vascular invasion, malignancy can be based upon the expression of Ret/PTC or CK-19 gene rearrangements. Considering Ki-67 as an endpoint marker of multiple pathways in cellular proliferation, Hoos *et al*. investigated its role in HCC (vascular invasion, capsular invasion and extrathyroid extension/dissemination). They found that Bcl-2 expression > 50% was associated with relapse-free survival and disease-specific survival. So Combination of Ki-67 (+) and Bcl-2 (−) phenotype was associated with widely invasive and aggressive HCC, compared to normal tissue. Also they found inactivation of p53 protein, but p21 overexpression in 43–63% of Hürthle cell tumours, suggesting the role of this protein in thyroid tumourogenesis.^7^

HCCs and follicular carcinomas express various oncogenes in different ways. In comparison with follicular carcinomas, HCCs express a greater proportion of Pan-ras, N-myc, transforming growth factor alpha (TGF-α), TGF-β and insulin-like growth factor 1 (IGF-1).^12^ So, these findings confirm that HCCs and follicular carcinomas are different entities and not only a subtype one of the other. Recent reports suggest that use of some proliferative cell markers such as Ki-67 or various oncogenes could be useful in distinguishing malignant and benign tumours.^13^

The main procedure of treatment for HCC is thyroidectomy. But a question still prevails about the optimal resection extent for HCC - total thyroidectomy versus lobectomy. Lobectomy could be adequate for smaller tumours (less than 1 cm). There are several advantages when total thyroidectomy is performed:

- In case of multifocal disease, which is found in about 35% of patients;
- Removal of thyroid gland facilitates radioactive iodine uptake in recurrent disease;
- Assessment of Tg is more sensitive for recurrent disease in absence of normal thyroid tissue.

Whenever HCC is diagnosed and thyroidectomy performed, any remaining tissue should be ablated with radioactive iodine. When thyroglobulin is still detectable after total thyroidectomy and ablation with radioiodine or when its value begins to rise, we should think that recurrence could be in question. The problem of distant metastases is still controversial. Treatment with radioactive iodine is of less beneficial results. According to the current guidelines for treatment of progressive or symptomatic HCC metastatic disease it is recommended:^14^

- Use of multikinase inhibitors (MKIs), especially pazopanib, sorafenib or suntinib;
- Systemic oncological therapeutic protocols;
- Best supportive care.

We report a case of this rare disease where appearance of HCC was documented extremely soon after both HCA and follicular thyroid adenoma were diagnosed on pathohistology of the removed thyroid tissue. This case is an example of rare and complex thyroid pathology concerning two aspects: firstly the concomitant existence of the two entities - follicular thyroid adenoma and HCA (with small cellular atypia), secondly because of the fast growing tumour, probably evolving from the micro multifocal HCC in the remnant thyroid tissue, left over after the surgical removal of HCA.

Knowing the possible multifocal nature of HCC, we assume that in this patient, we see very fast evolution and progression of the micro multicentric HCC in the remnant thyroid tissue. Therefore we verify immediate and last phase of malignant transformation.

HCC are slow progressive neoplasms, with slow evolution, involving a long period of existence and growing of a nodule with HCA and cellular atypia, as in our case (the first nodule in the left thyroid lobe reached a diameter of 5cm). Concerning the short period of malignant transformation (almost 1 year), as well as absence of any malignant cellular characteristics by FNAB (the well-known problem in the distinction between HCA and HCC), the fact of not developing any local metastasis in the neck is not a surprise.

FNAB is simple non-invasive technique for sampling thyroid palpable/non-palpable nodules, but it cannot rule out with certainty the possibility of presence of malignant focus in multifocal HCC. Scintigraphy with ^99m^Tc-MIBI helps in evaluating metabolic activity in the fast growing tumour (as in our case of HCA/HCC), rising a suspicion of malignant transformation and as a relative indication for surgical operation. WBS with this tracer is a good diagnostic modality for evaluation of distant metastases. Considering the multifocality of the disease with micro variant that was not seen on US, but happened to be present in the remnant thyroid tissue on the left side suggests the total thyroidectomy to be a procedure of first choice in these patients.

The fast growing rate, the size of the thyroid nodule (39 mm) and the presence of necrotic lesions and capsule invasion detected by histopathology on one side and not having the opportunity to evaluate the expression for Ret/PTC or CK-19 gene rearrangements and proliferation index Ki-67 on the other side, guided us to post-therapeutic procedure with 100 mCi ^131^I for achieving optimal conditions for further evaluation and disease treatment.

## Conclusions

The differences between HCAs and HCCs could be clearly made only with histopathological evaluation of the operated specimen, although there are some clinical features used to predict this difference, especially the new molecular markers. This case report confirms the fact that HCC should never be excluded whenever HCA is diagnosed, especially with cellular atypia.

Scintigraphy with ^99m^TcO_4_ usually presents “cold” nodule, and dual phase ^99m^Tc-MIBI scintigraphy presents “hot” nodule, so in case of HCA/HCC, combination of these two diagnostic modalities could improve the final clinical decision.

Patients with cytological diagnosis of HCNs should proceed to total thyroidectomy, especially if tumour size is > 1 cm, if FNAB findings comprise cellular atypias and / or multiple bilateral nodules are detected in the thyroid, as in our case. Total thyroidectomy allows follow up tests to be more effective thereafter, and also enables better disease prognosis by avoiding the possibility of recidivant HCC appearance.
